# The additive effect of late-life depression and olfactory dysfunction on the risk of dementia was mediated by hypersynchronization of the hippocampus/fusiform gyrus

**DOI:** 10.1038/s41398-021-01291-0

**Published:** 2021-03-17

**Authors:** Ben Chen, Xiaomei Zhong, Min Zhang, Naikeng Mai, Zhangying Wu, Xinru Chen, Qi Peng, Huarong Zhou, Qiang Wang, Mingfeng Yang, Si Zhang, Lavinia Alberi Auber, Ilona Croy, Thomas Hummel, Yuping Ning

**Affiliations:** 1 Department of Geriatric Psychiatry, The Affiliated Brain Hospital of Guangzhou Medical University (Guangzhou Huiai Hospital), Guangzhou, Guangdong Province China; 2 Smell and Taste Clinic, Department of Otorhinolaryngology, Technische Universität Dresden, Dresden, Germany; 3 Department of Neurology, The Affiliated Brain Hospital of Guangzhou Medical University (Guangzhou Huiai Hospital), Guangzhou, Guangdong Province China; 4 Department of Medicine, University of Fribourg, Fribourg, Switzerland; 5 Swiss Integrative Center of Human Health, Fribourg, Switzerland; 6 Department of Psychosomatic Medicine, Technische Universität Dresden, Dresden, Germany; 7 The first School of Clinical Medicine, Southern Medical University, Guangzhou, Guangdong Province China; 8 Guangdong Engineering Technology Research Center for Translational Medicine of Mental Disorders, Guangzhou, China

**Keywords:** Human behaviour, Diagnostic markers

## Abstract

Early detection of patients with late-life depression (LLD) with a high risk of developing dementia contributes to early intervention. Odor identification (OI) dysfunction serves as a marker for predicting dementia, but whether OI dysfunction increases the risk of dementia in LLD patients remains unclear. The present study aimed to explore the interactive effect of LLD and OI dysfunction on the risk of dementia and its underlying neuroimaging changes. One hundred and fifty-seven LLD patients and 101 normal controls were recruited, and data on their OI, cognition, activity of daily living (ADL), and resting-state functional magnetic resonance imaging were collected. Two × two factorial analyses were used to analyze the interactive effects of LLD and OI dysfunction on neuropsychological and neuroimaging abnormalities. Mediation analyses were used to explore whether abnormalities detected by neuroimaging mediated the the associations between OI and cognition/ADL. The results suggested that LLD and OI dysfunction exhibited additive effects on reduced ADL, global cognition and memory scores, as well as neuroimaging variables including (i) increased fractional amplitude of low-frequency fluctuation (fALFF) in the right orbitofrontal cortex and right precentral cortex, and (ii) increased regional homogeneity (ReHo) in the left hippocampus/fusiform gyrus, etc. In addition, these increased fALFF and ReHo values were associated with reduced neuropsychological scores (ADL, global cognition, memory, and language). Moreover, ReHo of the left hippocampus/fusiform gyrus completely mediated the relationship between OI and ADL, and partially mediated the relationship between OI and global cognition. Overall, mediated by the hypersynchronization of the left hippocampus/fusiform gyrus, OI dysfunction may increase the risk of dementia in LLD patients.

## Introduction

Late-life depression (LLD), which affects 3.5–7.5% of the geriatric population (over age 60 years), is a risk factor for dementia^1,2^. The risk of developing dementia in patients with LLD was 1.71–6.75 times higher than that in healthy elderly^3,4^. Therefore, early screening of patients with LLD with a high risk of developing dementia would be advantageous for timely intervention, improve their clinical outcomes, and reduce the morbidity of dementia. LLD shares many common mechanisms with Alzheimer’s disease (AD), such as metabolic disturbance in glucocorticoid steroids^5^, hippocampal dysfunction^6^, dysfunctions of brain-derived neurotrophic factors^7^, and neuroinflammatory changes^8^. These common mechanisms may accelerate neurodegeneration in patients with LLD and, as a result, increase their risk of conversion to AD. Hence, the assessment of amyloid β (Aβ) and tau by cerebrospinal fluid (CSF) or positron emission tomography–computed tomography (PET-CT) may contribute to the early detection of neurodegeneration in patients with LLD^9–11^. However, their invasiveness and high cost restrict them from being widely used. Hence, non-invasive markers that require less time and that are more cost-effective may facilitate the early detection of individuals with a high risk of developing dementia^12^.

Among the different kinds of olfactory dysfunction (such as odor threshold and odor memory), odor identification (OI) dysfunction is the strongest predictor of AD and the assessment of OI exhibits the advantages of non-invasiveness, cost-effectiveness, and high patient compliance^13^. In AD pathological progression, the emergence of OI dysfunction may be parallel to tau-mediated neuronal injury and occur 5–10 years earlier than memory impairment and clinical symptoms^13,14^. Other studies have suggested that OI dysfunction was associated with worse cognitive performance (memory, execution function, and language)^15^, reduced hippocampal and entorhinal volume^16^, increased cortical amyloid burden^17^, and lower ratios of CSF t- tau and P_181_-tau to Aβ_1-42_^18^. In addition, in community elderly individuals and patients with amnestic mild cognitive impairment, OI dysfunction predicts faster cognitive decline and a higher rate of conversion to AD^19^, in particular when combined with neuropsychological assessments and neuroimaging evaluations^20^. Conversely, intact global cognition and OI predicted a lack of transition to dementia in older adults^21^.

Whether OI dysfunction may contribute to the early detection of individuals with a high risk of dementia in patients with LLD remains unclear. Our previous results suggested that OI was positively correlated with cognitive performance, especially memory in LLD patients. In addition, compared with LLD patients with intact OI, LLD patients with OI dysfunction exhibited worse cognitive function and more structural brain abnormalities, and their pattern of structural abnormalities was similar to that of patients with AD^22^. However, the mechanisms by which OI dysfunction was associated with more severe cognitive impairment and structural abnormalities need to be further explored. Recent studies have demonstrated the close relationships among olfactory dysfunctions, depression, brain malfunction, and AD risk^23,24^. On one hand, when doing olfactory task, individuals with olfactory dysfunction would be expected to make more effort to perform normally compared with individuals with normal olfaction, resulting in hyperactivity in their olfactory sensory and cognitive processing areas^13,25^. Moreover, hyperactivity during effortful cognitive tasks was associated with structural abnormalities and Aβ deposition in individuals at the risk of AD^26^. On the other hand, many brain areas related to emotional and cognitive processing overlap with the olfactory pathway (such as the hippocampus, insula, and orbitofrontal cortex), and there is a reciprocal interaction between olfactory dysfunction and emotional dysfunction^27^. Therefore, changes in olfaction can affect depressive symptoms^28^ and depression can also lead to olfactory dysfunction^29^.

Based on the above evidence, we hypothesized that OI dysfunction may be associated with hyperactivity in overlapping brain regions involved in the processing of olfaction, depression and cognition, and facilitate functional abnormalities in patients with LLD. As a result, this hyperactivity would worsen their cognitive performance and activity of daily living (ADL). Therefore, by designing two × two factorial analyses, the present study aimed to explore whether there were additive effects between LLD and OI dysfunction on cognitive impairment and ADL disability, and on brain functional abnormalities. In addition, mediation analyses were used to explore whether the relationships between OI dysfunction and cognitive impairment and ADL disability may be mediated by functional abnormalities in the overlapping regions, and which specific parts of the overlapping regions may exhibit this mediated effect.

## Methods

### Subjects

One hundred fifty-seven LLD patients were continuously recruited from The Affiliated Brain Hospital of Guangzhou Medical University (Guangzhou Huiai Hospital) and 101 normal controls (NCs) were recruited from the communities in Guangzhou. All of the subjects were Han Chinese people. All of the subjects or their legal guardians offered written informed consent to take part in the study. This study was approved by the Ethics Committees of The Affiliated Brain Hospital of Guangzhou Medical University (2014, 078).

The inclusion criteria for patients with LLD were as follows: (1) age > 60 years; (2) met the criteria for major depression in the Diagnostic and Statistical Manual of Mental Disorders, Fourth Edition; and (3) clinical stage and diagnosis confirmed by trained physicians from our hospital. The NC exhibited normal cognitive function and had no history of depression. The exclusion criteria were as follows: (1) history of other major psychiatric disorders, such as bipolar disorder and schizophrenia; (2) family history of schizophrenia and bipolar disorders; (3) physical illness that may induce emotional abnormalities, such as anemia and hypothyroidism; (4) neurological disease, such as brain tumor and stroke; (5) current or previous psychotic symptoms; and (6) other situations that significantly influence olfaction, including active upper respiratory/sinus infection or respiratory distress at the time of testing, congenital or traumatic anosmia, known nasal polyps or tumors, current or recent (past 6 months) smoking, and alcohol or substance dependence. Diagnoses were made by at least two neurologists with dementia expertise, one neuropsychologist, and one geriatric psychiatrist.

### Neuropsychological assessments

Subjects were interviewed by neuropsychologists after finishing standard clinical assessments. Subjects underwent comprehensive neuropsychological tests, including an evaluation of depression using the 17-item Hamilton Depression Rating Scale (HAMD-17) (score range 0–52), ADL using the Activities of Daily Living Scale (ADL) score range 0–14), global cognition using the Mini-Mental State Examination (MMSE) (score range 0–30), memory using the recognition session of Auditory Verbal Learning Test (score range 0–24), language using the Verbal Fluency Test (the scores depend on how many names of animals are generated in 60 s), executive function using the Trail-Making Test B (the scores depend on how many seconds the subject takes to finish the test), attention using the Digit Span Test (score range 0–7) and visuospatial skills using the Rey-Osterrieth complex figure (score range 0–36).

### Olfactory assessments

OI was assessed using the Sniffin’ Sticks Screen 16 test battery^30^. Subjects finished a questionnaire that surveyed factors that may influence olfactory function (history of nasal trauma and nasal surgery, history of radiation or chemotherapy, difficulty breathing through one side of the nose, etc.). All olfactory assessments were carried out in a relatively odorless and ventilated room in The Affiliated Brain Hospital of Guangzhou Medical University. All of the subjects underwent OI tests following the neuropsychological assessments on the same day.

A score < 10 indicated the presence of OI dysfunction; after assessment, subjects were divided into four groups: LLD with OI dysfunction, LLD without OI dysfunction, NC with OI dysfunction, and NC without OI dysfunction^22,31^.

### MRI data acquisition

Subjects underwent magnetic resonance (MR) scans after neuropsychological and olfactory assessments within 3 days. The Philips 3.0T MR systems in The Affiliated Brain Hospital of Guangzhou Medical University (Philips, Achieva, The Netherlands) was used to acquire the imaging data. Fifty-four LLD patients with intact OI and 41 with OI dysfunction, as well as 47 NC subjects with intact OI and 35 with OI dysfunction, were included. For each participant, an anatomical image was obtained with a sagittal three-dimensional gradient echo T1-weighted sequence (TR = 8.2 ms, TED = 3.8 ms, TI = 1100 ms, flip angle (FA) = 8°, 188 slices, slice thickness = 1 mm, gap = 0 mm, matrix = 256 × 256, and inversion time = 0). Sagittal resting-state functional MRI (fMRI) datasets of the whole-brain were obtained in 6 min with a single-shot gradient echo-planar imaging pulse sequence. The resting-state fMRI scanning parameters were as follows: TE = 30 ms, TR = 2000 ms, FA = 90°, number of slices = 33, slice thickness = 4 mm, matrix size = 64 × 64, and field of view = 220 × 220 mm.

### Image processing

Resting-state fMRI data pre-processing was carried out using the Data Processing Assistant for Resting-State 4.5 (DPASF 4.5)^32^. The first ten volumes were removed to preserve steady-state data only. The remaining images were corrected for timing differences and for head motion. Subjects who had images with more than 2 mm translational movement or more than 2° rotational movement were excluded from further analysis. The individual structural image (T1-weighted image) was co-registered to the mean functional image after motion correction. The transformed structural images were segmented into gray matter, white matter, and CSF. Nuisance signals, such as six head motion parameters, the global signal, the CSF signal, and the white matter signal, were regressed out from each time series. Following this, the motion-corrected functional images were spatially normalized into Montreal Neurological Institute space and resampled to 3 × 3 × 3 mm^2^ using the normalization parameters estimated during unified segmentation. Subsequently, the functional images were smoothed with a 6 mm full width at half-maximum (FWHM) Gaussian kernel and detrending was carried out. Finally, a bandpass filter (0.01 Hz < *f* < 0.1 Hz) was applied to reduce the effect of low-frequency drifts and high-frequency noise^33^.

### Analyses of fractional amplitude of low-frequency fluctuation and regional homogeneity

The fractional amplitude of low-frequency fluctuation (fALFF)^34^ and regional homogeneity (ReHo)^35^ values were calculated by DPASF 4.5. The sum of the absolute amplitudes of low frequency (across from 0.01 to 0.08 Hz) was extracted from the time series of each voxel, which was defined as the ALFF and reflected spontaneous neuronal activities. Extended from the ALFF, the fALFF was introduced to overcome the limitations of ALFF, such as the signal fluctuations that are elicited by physiological noise. The fALFF represents the fractional sum of the amplitudes at low frequency divided by the entire frequency range (from 0 to 0.25 Hz)^34^. ReHo reflects the degree of local regional neural activity coherence. Briefly, it was calculated as Kendall’s coefficient of concordance (or Kendall’s W) of the time course of a given voxel with those of its nearest neighbors (26 voxels). For the purpose of standardization, the ReHo value of each voxel was divided by the global mean ReHo value. Finally, the resulting ReHo images were spatially smoothed with a 6 mm FWHM Gaussian kernel^35^.

### Statistics

Statistical Package for Social Sciences version 22.0 (IBM SPSS 22.0, Chicago, IL, USA) was used to perform the statistical analyses. Demographic and clinical variables among groups were evaluated with *χ*^2^ analysis and one-way analysis of variance.

Factorial analysis was used to analyze the influence of LLD and OI dysfunction on neuropsychological indicators. Factors were divided into different levels. The factor “Depression” included two levels: LLD and NC. The factor “Olfaction” included two levels: OI dysfunction and Intact OI. Control variables included age, sex, and years of education. Least significant difference post hoc analysis was used for multiple comparisons. Factorial analysis with the same design was used to analyze the influence of “Depression” and “Olfaction” on fALFF and ReHo values, and control variables included age, sex, years of education, and gray matter images. Correction for multiple comparisons was performed using a false discovery rate at *P* < 0.05. Partial correlation analyses were used to analyze the associations between neuropsychological scores and neuroimaging indicators, which had been shown to be significant in factorial analysis. Control variables included age, sex, and years of education.

Mediation analyses were performed for potential variables screened in factorial analysis. The mediation model was established when the following conditions were met: (1) the independent variable (IV) had a significant effect on the dependent variable (DV); (2) the IV significantly predicted the mediator; (3) the mediator significantly affected the DV; and (4) when the mediator was excluded in the model, the effect of the IV on the DV decreased. In our analysis, OI scores were regarded as the IV, neuropsychological indicators were regarded as DVs, and neuroimaging indicators were regarded as mediators. PROCESS 3.2 was used to investigate the mediation model among variables^36^. Indirect effects were estimated with 1000 bootstrapped samples. Moreover, the Sobel test was performed to verify whether the mediating effect was significant^37,38^.

## Results

### Additive effect of LLD and OI dysfunction on cognitive impairment and ADL disability

The demographic, olfactory, and cognitive information of all subjects is listed in Table 1. Significant differences were found in OI, HAMD, ADL, and all aspects of cognitive function among the four groups.

**Table 1:** Demographic, olfactory, and neuropsychological information of all subjects.

	NC with intact OI (*n* = 70)	NC with OI dysfunction (*n* = 31)	LLD with intact OI (*n* = 93)	LLD with OI dysfunction (*n* = 64)	*F*	*P*
Age	66.5 ± 6.3	69.6 ± 5.8	66.6 ± 7.1	68.5 ± 7.4	2.019	0.112
Male/female	25/45	15/16	19/74	19/45	10.087	0.018
Years of education	10.8 ± 3.1	11.9 ± 3.8	9.1 ± 3.8	8.3 ± 3.8	8.688	<0.001
OI	12.1 ± 1.3	7.9 ± 1.9	11.8 ± 1.1	7.1 ± 1.7	81.793	<0.001
HAMD	2.2 ± 2.7	1.9 ± 2.7	8.9 ± 7.6	10.0 ± 7.7	14.310	<0.001
ADL	13.9 ± 0.6	14.0 ± 0	13.7 ± 0.6	12.9 ± 2.8	4.445	<0.001
Global cognition	26.7 ± 2.3	27.6 ± 1.6	24.3 ± 4.0	20.1 ± 6.5	22.013	<0.001
Memory	21.9 ± 1.7	21.7 ± 1.5	21.0 ± 2.7	18.9 ± 3.7	13.002	<0.001
Language	11.3 ± 3.6	11.5 ± 2.9	8.6 ± 3.2	7.7 ± 3.8	6.143	<0.001
Executive function (seconds)	58.7 ± 24.0	60.5 ± 15.6	82.1 ± 36.2	96.3 ± 38.6	15.334	<0.001
Visual–spatial skill	27.9 ± 4.1	26.0 ± 5.5	23.6 ± 6.3	22.3 ± 8.8	12.486	<0.001
Attention	5.5 ± 1.0	5.5 ± 0.9	5.1 ± 1.2	4.5 ± 1.2	8.761	<0.001

*ADL* Activities of Daily Living Scale, *HAMD* Hamilton Depression Rating Scale, *LLD* late-life depression, *NC* normal controls, *OI* odor identification.

**Fig. 1:**
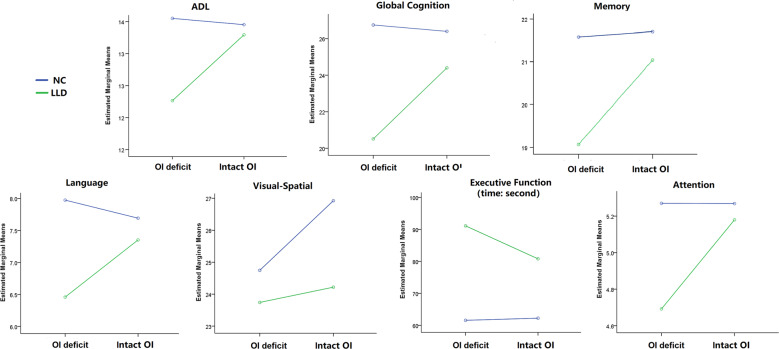
Additive effects of LLD and OI dysfunction on cognitive impairment and ADL disability. Two × two factorial analyses were used to analyze the influence of the factor “Depression” (LLD and NC) and the factor “Olfaction” (OI dysfunction and intact OI) on neuropsychological scores; control variables included age, sex, and years of education. LLD, late-life depression; NC, normal controls; OI, odor identification.

In factorial analyses, the factor “Depression” showed significant effects on scores of ADL, global cognitive function, memory, executive function, and attention (LLD < NC). The factor “Olfaction” showed significant effects on scores of ADL, global cognitive function, and memory (intact OI > OI dysfunction). In addition, there were interactive effects between the factors “Depression” and “Olfaction” on reduced scores of ADL, global cognition, and memory. Furthermore, LLD patients with OI dysfunction exhibited lower scores than LLD patients with intact OI and two NC groups in the post hoc analyses (*P* < 0.05) (Fig. 1 and Table 2).

**Table 2:** Additive effects of LLD and OI dysfunction on cognitive impairment and ADL disability.

	Depression		Olfaction		Interaction	
	*F*	*P*	*F*	*P*	*F*	*P*
ADL	9.841	0.002	4.425	0.037	6.611	0.011
Global cognition	40.903	<0.001	8.202	0.005	11.960	0.001
Memory	15.215	<0.001	7.156	0.008	5.636	0.018
Language	5.633	0.019	0.660	0.418	2.500	0.115
Executive function	24.366	<0.001	1.052	0.305	1.395	0.238
Visual–spatial skill	3.753	0.054	2.071	0.151	0.871	0.351
Attention	3.965	0.048	2.283	0.132	2.369	0.126

*ADL* Activities of Daily Living Scale.

### Additive effect of LLD and OI dysfunction on brain functional abnormalities

**Fig. 2:**
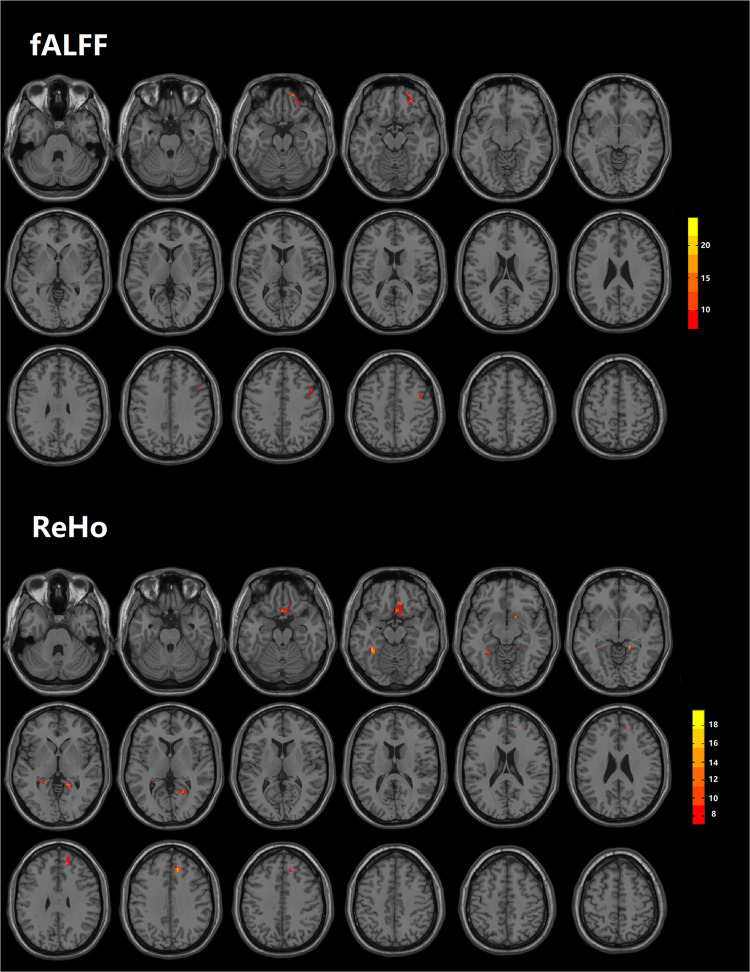
Additive effects between LLD and OI dysfunction on fALFF and ReHo. Two × two factorial analyses were used to analyze the influence of the factor “Depression” (LLD and NC) and factor “Olfaction” (OI dysfunction and intact OI) on fALFF and ReHo, and control variables included age, sex, years of education, and gray matter images. Multiple comparison correction was performed using a false discovery rate (FDR) at *P* < 0.05. The color scale bar shows the logarithmic scale of *p*-values (−log10). The closer to yellow, the more significant the difference between groups. fALFF, fractional amplitude of low-frequency fluctuation; ReHo, regional homogeneity.

In the analyses of fALFF, LLD and OI dysfunction showed additive effects on increased fALFF values in the right orbitofrontal cortex and right precentral cortex (Table 3 and Fig. 2). Neither “Depression” nor “Olfaction” exhibited significant independent effects on fALFF. In the analyses of ReHo, LLD and OI dysfunction showed additive effects on increased ReHo in the right rectus/entorhinal cortex (cluster size 23/20), left hippocampus/fusiform gyrus (cluster size 25/12), right calcarine/lingual/parahippocampal gyrus (cluster size 13/10/10), and right superior frontal gyrus. Neither “Depression” nor “Olfaction” exhibited a significant independent effect on ReHo (Table 3 and Fig. 2). The significant regions found in factorial analyses were defined as “additive regions.”

**Table 3:** Additive effects between LLD and OI dysfunction on brain hypersynchronization.

	Brain regions	Peak MNI			Cluster size	*F*
		*x*	*y*	*z*		
fALFF	Right orbitofrontal cortex	27	45	−18	29	17.670
	Right precentral cortex	39	3	42	17	24.392
ReHo	Right rectus/entorhinal cortex	3	18	−18	59	14.093
	Left hippocampus/fusiform gyrus	−33	−48	−12	43	18.035
	Right Calcarine/lingual/parahippocampal gyrus	18	−39	−6	39	13.590
	Right superior frontal gyrus	12	36	36	38	15.838

*fALFF* fractional amplitude of low-frequency fluctuation, *MNI* Montreal Neurological Institute, *ReHo* regional homogeneity.

### Associations between functional activities of additive regions and neuropsychological scores

**Fig. 3:**
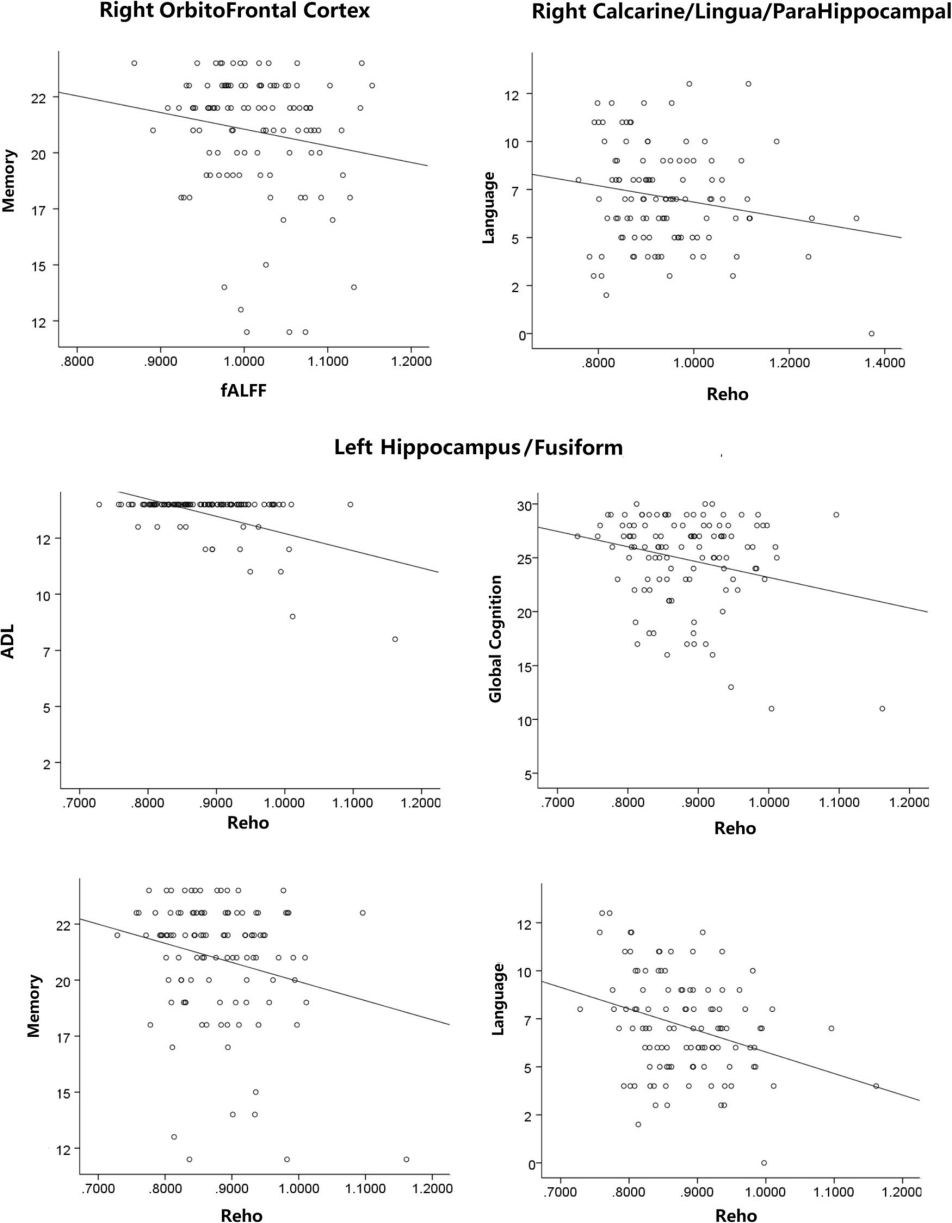
Correlations between cognitive function and brain activities in additive regions across all subjects. ADL, Activities of Daily Living Scale; fALFF, fractional amplitude of low-frequency fluctuation; ReHo, regional homogeneity.

Across all subjects, partial correlation analyses showed that ReHo of the left hippocampus/fusiform gyrus was associated with ADL (*r* = −0.394, *P* < 0.001), global cognition (*r* = −0.194, *P* = 0.044), memory (*r* = −0.215, *P* = 0.026), and language (*r* = −0.285, *P* = 0.003); ReHo of the right calcarine/lingual/parahippocampal gyrus was associated with language (*r* = −0.201, *P* = 0.039); and fALFF of the right orbitofrontal cortex was associated with memory (*r* = −0.207, *P* = 0.031) (Fig. 3). No significant result was found among the other three additive regions and neuropsychological scores (*P* > 0.05).

### Associations between OI and neuropsychological scores were mediated by ReHo of the left hippocampus/fusiform gyrus

**Fig. 4:**
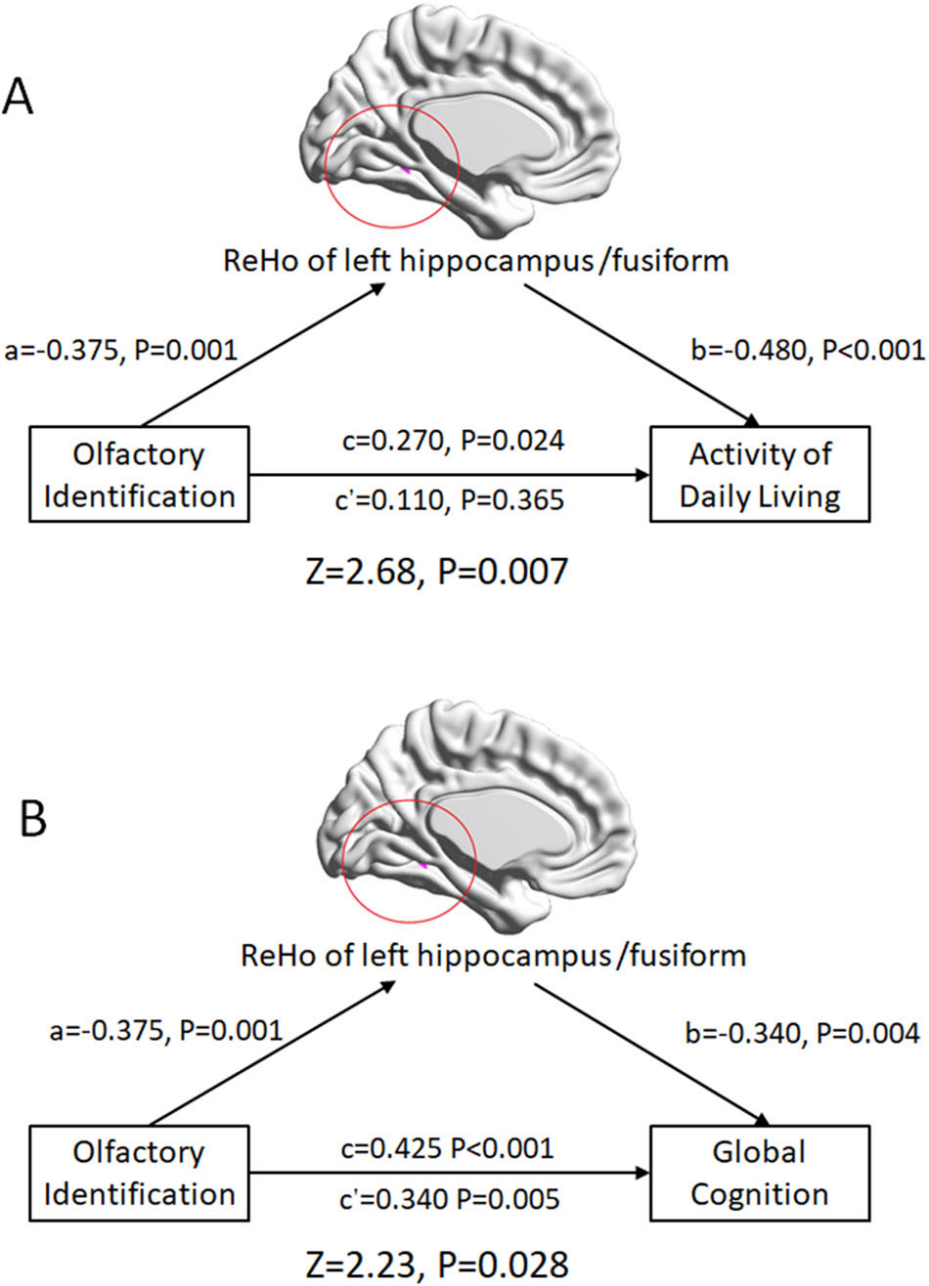
Associations between OI and global cognition/ADL were mediated by ReHo of the left hippocampus/fusiform gyrus. The ReHo of the left hippocampus/fusiform gyrus was **A** a complete mediator of the relationship between OI and ADL, and **B** a partial mediator of the relationship between OI and global cognition.

**Fig. 5:**
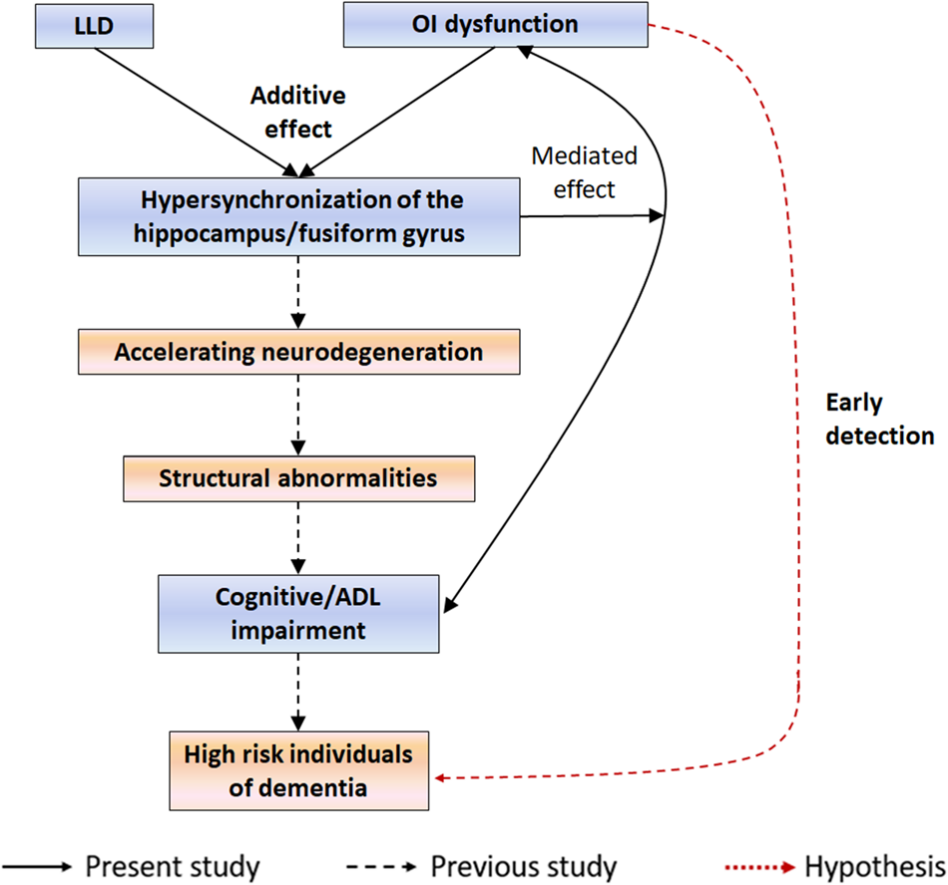
Potential mechanism of how OI dysfunction increases the dementia risk of patients with LLD. OI dysfunction and LLD exhibited additive effects that resulted in hypersynchronization of the hippocampus/fusiform gyrus, which is a region related to olfaction, depression, and cognition. Persistent hypersynchronization may facilitate neurodegeneration and cause structural abnormalities, leading to cognitive impairment and ADL disability. In addition, the associations between OI dysfunction and cognitive impairment and ADL disability were mediated by hyperactivity of the hippocampus/fusiform area. Overall, OI dysfunction may contribute to the early detection of individuals at high risk of dementia among patients with LLD. The blue box indicates the present analyses and the red box indicates future directions.

The total effect of OI on the ADL score was *β* = 0.270 (*t* = 2.307, *p* = 0.024), which manifested a significant positive effect of OI on ADL. The indirect effect of OI on ADL through ReHo of the left hippocampus/fusiform gyrus was −0.160 (*z* = 2.68, *p* = 0.007), which indicated that ReHo of the left hippocampus/fusiform gyrus as a mediator annulated the effect of OI on ADL. Furthermore, the remaining direct effect of OI on ADL was not significant *β* = 0.110 (*t* = 0.916, *P* = 0.365), with the effect of OI on ReHo of the left hippocampus/fusiform gyrus being *β* = −0.375 (*t* = −3.353, *p* = 0.001) and the effect of ReHo of the left hippocampus/fusiform gyrus on ADL being *β* = −0.480 (*t* = −4.445, *p* < 0.001). In summary, the above results showed that the ReHo of the left hippocampus/fusiform gyrus was a complete mediator of the relationship between OI and ADL (Figs. 4A and 5).

The total effect of OI on the global cognition score was *β* = 0.425 (*t* = 3.787, *p* < 0.001), which manifested a significant positive effect of OI on global cognition. The indirect effect of OI on global cognition through ReHo of the left hippocampus/fusiform gyrus was −0.085 (*z* = 2.23, *p* = 0.028), which indicated that ReHo of the left hippocampus/fusiform gyrus as a mediator annulated the effect of OI on global cognition. Furthermore, the remaining direct effect of OI on global cognition was still significant at *β* = 0.340 (*t* = 2.898, *p* = 0.005), with the effect of OI on the ReHo of left hippocampus/fusiform gyrus being *β* = −0.375 (*t* = −3.353, *p* = 0.001), and the effect of the ReHo of left hippocampus/fusiform gyrus on global cognition being *β* = −0.340 (*t* = −2.944, *p* = 0.004). In summary, the above results showed that ReHo of the left hippocampus/fusiform gyrus was a partial mediator of the relationship between OI and global cognition (Fig. 4B).

No significantly mediated effect was found in other neuroimaging indicators on the association between OI dysfunction and ADL/cognitive impairment.

## Discussion

The present study explore the interactive effect between LLD and OI dysfunction on cognitive impairment, and ADL disability and brain functional abnormalities. In addition, mediation analysis was used to explore the mediated effect of brain functional abnormalities on the relationships between OI dysfunction and cognitive impairment and ADL disability. Our results suggested that there were additive effects between LLD and OI dysfunction that resulted in (1) reduced scores of ADL, global cognition, and memory; (2) increased fALFF in the right orbitofrontal cortex and right precentral cortex; and (3) increased ReHo in the right rectus/olfactory cortex, left hippocampus/fusiform gyrus, right calcarine/lingual/parahippocampal gyrus, and right superior frontal gyrus. Among these neuroimaging variables, ReHo of the left hippocampus/fusiform gyrus exhibited a complete mediated effect on the relationship between OI and ADL, and exhibited a partially mediated effect on the relationship between OI and global cognition.

Previous studies have demonstrated that both LLD and OI dysfunction are risk factors for dementia^4,14^, but whether comorbidities of LLD and OI dysfunction may be associated with higher risk remains unclear. The present results suggest that there were additive effects between LLD and OI dysfunction that resulted in reduced scores of ADL, global cognition, and memory, suggesting that LLD patients with OI dysfunction exhibited more severe impairment of cognitive performance and ADL. It has been repeatedly reported that OI dysfunction could serve as a marker for indicating early AD pathology and predicting conversion to AD in community elderly individuals and patients with MCI^4^, and the assessment of OI has the advantages of non-invasiveness, cost-effectiveness, and high patient compliance. The results of the present study suggested that OI may also contribute to the early screening of individuals at high risk of dementia among patients with LLD. Follow-up studies are needed to confirm whether OI dysfunction can predict a higher rate of conversion to AD in patients with LLD. In addition, assessments of Aβ by CSF biomarkers or PET-CT are needed to explore whether LLD patients with OI dysfunction suffer from more pronounced neurodegeneration than LLD patients with intact OI.

The present analyses of resting-state fMRI suggested that there were also additive effects between LLD and OI dysfunction on increased fALFF and ReHo. As fALFF reflects spontaneous neuronal activities^34^ and ReHo reflects the degree of local regional neural activity coherence^35^, the current results indicated that LLD with OI dysfunction may be associated with more functional hyperactivity in the brain. Hyperactivity has been regarded as a compensatory mechanism in individuals with olfactory dysfunction, because these people are expected to expend more effort to perceive odors and process olfactory information^13,25,39^. Our results suggested that in LLD with OI dysfunction, this compensatory mechanism also worked in the resting state and could reflect contingent hyperactivity of these regions. However, there was no independent effect of OI dysfunction or LLD on brain functional abnormalities. This was not consistent with previous studies in which patients with LLD exhibited significantly abnormal ALFF^40,41^ and ReHo^42,43^ values, and OI dysfunction was associated with alterations in ReHo in Parkinson’s disease^44^. The lack of an independent effect of LLD or OI dysfunction on functional abnormalities in the current study may be due to two reasons as follows: (1) the slight imbalance in sample size among the four groups in factorial analyses may influence the statistical power and (2) there may be hyperactivity in some other regions that may not reach the level of significance, because the current multiple comparison corrections were relatively strict and many regions were eliminated after correction.

Murphy hypothesized that regarding the potential mechanism of olfactory impairment in AD, when a patient with olfactory dysfunction tries to identify or remember an odor, hyperactivity in olfactory sensory and cognitive processing areas occurs. Over the person’s lifespan, neurodegeneration progresses and causes structural abnormalities in the brain, resulting in impairment in cognition/ADL and accelerating conversion to AD^13^. In accordance with this hypothesis, the present results demonstrated that increased fALFF and ReHo in the additive regions were negatively correlated with ADL scores and cognitive function, confirming that hyperactivation and hypersynchronization were associated with ADL disability and cognitive impairment. Follow-up studies are needed to further explore the causal relationship between OI dysfunction, neurodegeneration, and functional and structural abnormalities.

In regard to the additive regions, most of them were not only important parts of the olfactory pathway but were also related to emotional and cognitive processing. Therefore, abnormalities of these regions may spontaneously be associated with olfactory, emotional, and cognitive dysfunction. Among these additive regions, the ReHo of the left hippocampus/fusiform gyrus was the only variable that exhibited mediated effects on the relationship between OI dysfunction and ADL/cognitive impairment, suggesting it had the most important role in regulating the interaction between olfaction, depression, and cognition. The hippocampus is an important part of the secondary olfactory cortex, which encodes olfactory information and stores olfactory memory^45^. In addition, the hippocampus is among the first of the brain areas to be altered in AD, and the extent of the abnormalities may reflect disease severity. Moreover, the hippocampus also plays a key role in the neurobiology of depression, because it is at the core of the hypothalamic-pituitary-adrenal axis. Therefore, structural and functional abnormalities of the hippocampus have been found in patients with olfactory dysfunction, depression, and cognitive impairment^24^. Located between the inferior temporal gyrus and the parahippocampal gyrus, the fusiform gyrus is also one of the earliest regions to be affected in AD^46^. The fusiform gyrus is involved in many aspects of cognition, such as processing color information, face and body recognition, within-category identification, and word recognition^47^. In addition, hyperactivity of the fusiform gyrus was associated with an overreaction to negative stimuli, more severe depressive symptoms and negative cognitive bias in patients with depression^48^. With respect to olfaction, the fusiform gyrus is involved in encoding chemosensory cues, adjusting OI and odor recognition^48^. A reduced volume of the fusiform gyrus was found in patients with olfactory dysfunctions and improving olfactory function with olfactory training may increase its volume^49^.

In the present mediation analyses, the ReHo of the left hippocampus/fusiform gyrus was a complete mediator of the relationship between OI and ADL scores, suggesting that ADL was not influenced by OI directly in patients with LLD but through the mediation of hypersynchronization of the hippocampus/fusiform gyrus. In addition, the ReHo of the left hippocampus/fusiform gyrus was a partial mediator of the relationship between OI and global cognition. On one hand, global cognition was mediated by hypersynchronization of the hippocampus/fusiform gyrus in LLD patients, which was similar to the ADL outcome. On the other hand, it suggested that there was a direct relation between OI and global cognition in LLD. Memory recall, denomination, attention, and many cognitive processes are required during the assessment of OI, which appears similar to the activity during the MMSE test^50^. Therefore, OI performance may be a parallel indicator of cognitive performance, despite the mediated effect through brain hypersynchronization. Overall, as impairment of ADL and global cognition are important symbols of dementia risk, the present study suggests that the increasing risk of dementia in LLD patients with OI dysfunction may result from two pathways as follows: (1) OI dysfunction causing hypersynchronization in overlapping regions facilitating cognitive impairment and ADL disability, and (2) parallel OI dysfunction representing poor performance in cognitive assessments.

There were several limitations in the present study. First, as OI dysfunction in NC is not as common as in LLD patients, the unbalanced sample sizes in the four groups in factorial analyses may influence the statistical power. Second, the present conclusion should be interpreted with caution, because the fALFF and ReHo in resting-state fMRI only represent a functional association but do not infer causality among brain dysfunction. Future studies using analyses of dynamic causal models and Granger causality analysis or using olfactory task-fMRI could further clarify the causal relationships among functional abnormalities, cognitive impairment and ADL disability, and OI dysfunction in patients with LLD. In addition, fALFF and ReHo values represent local abnormalities and future studies using network analyses could provide a deeper understanding of connectivity level. Third, the current results were derived from cross-sectional analyses and there was no direct evidence for neurodegeneration in patients with LLD. Follow-up studies are needed to confirm whether OI dysfunction can predict conversion to AD in patients with LLD. Fourth, patients with Lewy body dementia and Parkinson’s disease also exhibit cognitive impairment, depression, and olfactory deficit, which may be confounding factors of the present study. Future studies assessing Aβ by using CSF biomarkers or PET-CT will improve the specificity of using OI dysfunction to predict AD in LLD patients. Last, the present study did not exclude the possible effect of drugs, because many patients with LLD were taking variable doses of antidepressant medication.

In summary, the present study shows that LLD and OI dysfunction exhibited additive effects on the impairment in ADL and cognitive function, as well as hyperactivity in olfactory/depression/cognition-associated regions. In addition, hypersynchronization of the left hippocampus/fusiform gyrus mediates the relationship between OI dysfunction and the risk of dementia in patients with LLD. Finally, in patients with LLD, OI dysfunction may serve as a potential marker for the early screening individuals at high risk of dementia, facilitating early intervention (Fig. 5). Future studies are needed to further explore whether OI dysfunction could predict conversion to dementia in patients with LLD and the underlying mechanism of how OI dysfunction could predict dementia.
